# Worldwide inverse correlation between Bacille Calmette-Guérin immunization and COVID-19 morbidity and mortality

**DOI:** 10.21203/rs.3.rs-42927/v1

**Published:** 2020-07-16

**Authors:** Willis X. Li

**Affiliations:** University of California San Diego

**Keywords:** SARS-CoV-2, COVID-19, Bacille Calmette-Guérin (BCG)

## Abstract

The COVID-19 pandemic has spread to all countries in the world after more than six months since it was first reported in late 2019, and different countries have been impacted differently. Correlation analysis between COVID-19 death numbers and different demographic and socioeconomic factors for all world countries (n=210) as of June 1, 2020, reveals that COVID-19 deaths per million population in a country significantly correlates with the country’s median age (r=0.48, p=4.8e-4) and per capita gross domestic product (GDP) (r=0.55, p=4.14e-5), and inversely correlates with the country’s Bacille Calmette-Guérin (BCG) vaccination rate (r=−0.63, p=9.9e-7). COVID-19 death is found not significantly associated, however, with a country’s policy stringency index, population density, extreme poverty rate, hospital beds availability per thousand people, and diphtheria-tetanus-pertussis (DTP3) immunization. Old age is likely a confounding factor for the correlation between COVID-19 and per capita GDP (r=0.66, p=2.3e-7). To control for possible confounding effects of age, countries with similar median age were grouped and analyzed. The inverse correlation between BCG vaccination rates and COVID-19 case (r=−0.338, p=0.0082) and death (r=−0.411, p=0.0011) remained significant among the top 61 countries with highest median age. The current study suggests that BCG might be protective against SARS-CoV-2 infection.

## Introduction

Since first reported in Wuhan, China, in December 2019, the COVID–19 pandemic has reached all countries in the world by June 2020, and most countries seem to have passed their peak^1^. As of June 1, 2020, there have been more than 6.3 million total accumulated cases and 377,428 total deaths world wide due to COVID–19, with a fatality rate of 6%^1^. However, different countries have been impacted vastly differently, with more than a quarter of all cases and deaths happening in the United States and certain other countries recording few cases and no death. The differential impact of COVID–19 on different countries in the world begs the question of what factors are important in influencing COVID–19 morbidity and mortality.

As an infectious respiratory disease transmitted through human contacts, COVID–19 should have higher impact in countries with high population density, with low disease control stringency, and in countries that lack medical resources such as physicians and hospital beds, which are often associated with low per capita GDP. In addition, there have been observations of COVID–19 association with climate, BCG immunization, and various socioeconomic conditions of countries. Some of the observations were made in the early stages of the disease transmission when COVID–19 data are still accumulating, hindering the ability to draw meaningful conclusions. Now more than six months have passed since COVID–19 started and all countries have been affected, it is time to examine its transmission again and to identify factors that are most important for COVID–19 morbidity and mortality. Correlation studies with current data available for all countries in the world indicate that COVID–19 morbidity and mortality show strong inverse association with BCG immunization, which is statistically significant and is independent of age, another factor important for COVID–19 susceptibility. The study suggests that BCG vaccination may have protective effects against COVID–19.

## Results

### Disproportional distribution of COVID–19 cases and deaths in world countries

**Figure 1:**
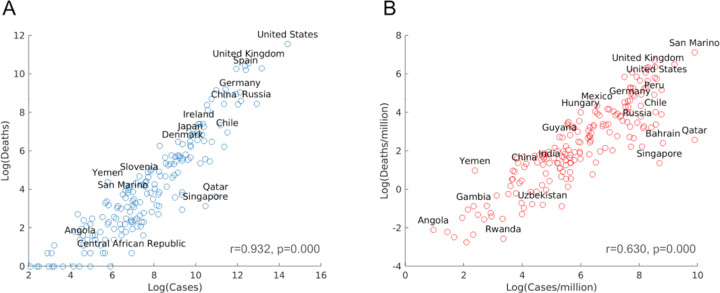
(A) Scatter plots in log scale of COVID-19 cases and deaths. (B) Scatter plots in log scale of COVID-19 cases and deaths per million. Only a subset of countries is labeled above its marker.

Although the COVID–19 pandemic has spread to all countries in the world, different countries have been impacted differently, based on data as of June 1, 2020. The U.S. recorded more than 2 millions cases and more than 100,000 deaths due to COVID–19, whereas some other countries these numbers are much lower, as shown by a scatter blot (Figure 1A). After controlling for population size, disparity for countries still remains, though a few countries with small population sizes, such as San Marino, lead the chart in COVID–19 cases and deaths per million population (Figure 1B). The case and death numbers are highly correlated, i.e., in general the higher the case number, the higher the death number in a country, although there are outliers with high case number but low death number, such as Qatar and Singapore (Figure 1). The unequal distribution of COVID–19 death and case numbers in different countries raises the question of what factors are important for the susceptibility of a population to SRAS-CoV–2 infection.

### Demographic and socioeconomic factors associated with COVID–19

To understand what demographic or socioeconomic factors are important for the COVID–19 impact, a pairwise correlation analysis was carried out for several factors with data available for most countries. These factors include each country’s total deaths per million due to COVID–19, government response stringency index, which records the strictness of ‘lockdown style’ policies that primarily restrict people’s behavior^2^, population density, median age, per capita gross domestic product (GDP), extreme poverty index, hospital beds per thousand people, the coverage rates of Bacille Calmette-Guérin (BCG) vaccination, and diphtheria-tetanus-pertussis (DTP3) immunization rates. BCG is a vaccine against tuberculosis (TB), but there has been observations of its correlation with COVID–19 impact^3^. DTP3 is a combined vaccine offered to young children in many, but not all countries with historical data available similarly to those of BCG immunization^4^. Since COVID–19 death and case numbers are highly correlated, only total deaths per million was included in the correlation analysis to reduce complexity.

**Figure 2:**
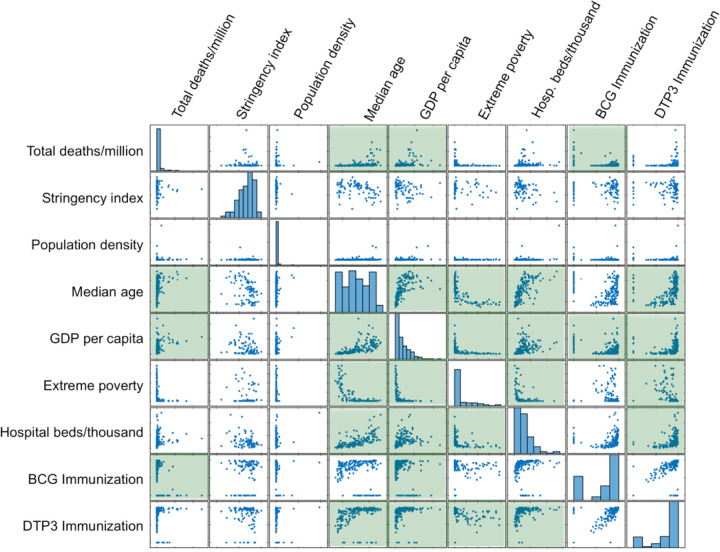
Pairwise scatter plots of the indicated independent variables. Green shaded regions indicate p<0.05 by Pearson’s correlation coefficient (see Table 1 for values).

When these factors were pairwise analyzed as independent variables using Pearson’s correlation coefficient, it is found that COVID–19 death per million is significantly positively associated with a country’s median age (r = 0.48, p = 4.8e–4) and with per capita GDP (r = 0.55, p = 4.14e–5), and is significantly negatively associated with the country’s BCG vaccination coverage rates (r = −0.63, p = 9.9e–7) (Figure 2, Table 1). COVID–19 death is found not significantly associated, however, with the country’s government response stringency index, population density, and hospital bed availability. COVID–19 death also has an inverse correlation with country’s poverty, although with only borderline significance (r = −0.28, p = 0.0539) (Figure 2, Table 1). Thus, more COVID–19 deaths are found in countries with higher per capita GDP and in countries with the more elderly people, whereas fewer COVID–19 deaths were found in countries with higher rates of BCG vaccination.

### Correlation between COVID–19 death and GDP may be due to age

It has been reported that COVID–19 affects older people more than younger people^5^. Indeed, COVID–19 deaths show highly significant positively association with the median age of nations (r = 0.48, p = 4.8e–4). On the other hand, the median age of a country highly significantly correlates with its per capita GDP (r = 0.66, p = 2.3e–7). It is thus likely that a high per capita GDP leads to longer lifespan, thereby raising the median age of a country. In other words, the higher case and death numbers in wealthy countries were more likely due to higher age as a confounding factor in those countries.

### COVID–19 death significantly inversely correlates with BCG but not DTP3 immunization

BCG is a TB vaccine used in many countries with high TB prevalence^3^. But it is not generally used in countries with low risks of TB infection, such as the US and most Western European countries. There have been reports of COVID–19 and BCG association with both positive and negative results^6–8^, and there are ongoing clinical trials using BCG as vaccine against COVID–19^9,10^.

**Figure 3:**
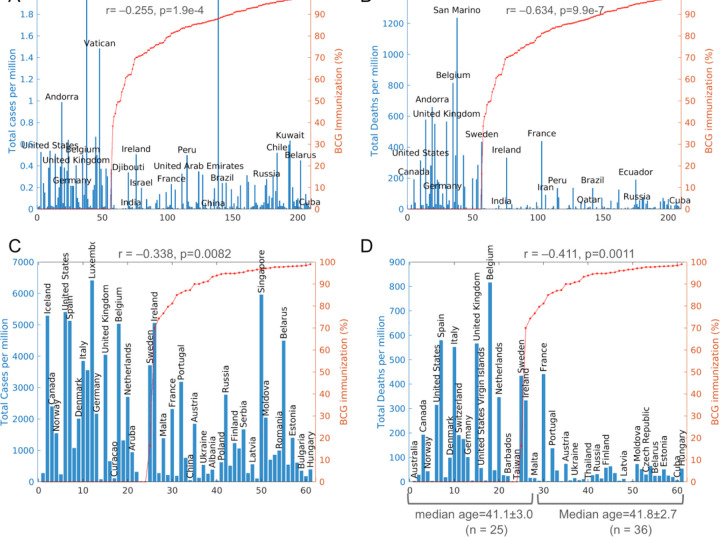
COVID-19 morbidity and mortality vs BCG immunization rates (A, B) Total COVID-19 cases and deaths per million against BCG immunization rates for all countries and regions (n=210) in the world. Only select countries are labeled. (C, D) Total COVID-19 cases and deaths per million against BCG immunization rates for high median age (“old”) countries and regions (n=61). r and p are from Pearson’s correlation coefficient analysis.

The average BCG vaccination rates over all the years show highly significant inverse correlation with COVID–19 deaths (Figure 2, Table 1). Indeed, when countries are sorted by mean BCG immunization rate, it can be seen that most of the high COVID–19 case and death numbers occurred in countries that do not adopt universal BCG vaccination policy (Figure 3A, B).

To understand if another type of vaccination also influences COVID–19 case and death, immunization diphtheria tetanus toxoid and pertussis (DTP3) was analyzed. DTP3 immunization is another long running vaccine program adopted non-uniformly by different countries, and broad immune benefits have been reported for both BCG and DTP3^11,12^. However, unlike BCG, no significant correlation was found between DTP3 immunization and COVID–19 death (r = 0.236, p = 0.100; Figure 2, Table 1). Thus, BCG vaccination might have specific effects against SARS-CoV–2 infection.

### BCG vaccination and COVID–19 death inversely correlate in countries with high median age

Since age is a significant confounder in the association between GDP and COVID–19, is age similarly a confounding factor in the correlation between BCG vaccination and COVID–19? Correlation coefficient analysis shows that BGC vaccination is not significantly associated with a country’s median age, although the two are inversely correlated (r = −0.2612, p = 0.0698; Figure 2), suggesting a trend that countries with lower BCG vaccination rates have a higher median age, thus raising the question whether the higher COVID–19 case and death numbers in countries lacking BCG vaccination can be explained by their having more elderly population.

To reduce the possible confounding effects of age, the correlation between BCG vaccination and COVID–19 was further analyzed by separating countries into three groups each with similar ages. The median ages of the world’s 210 countries range from 15.1 (Niger) to 48.2 (Japan) (Table 2). These countries were discretized into “young”, “medium”, and ‘old” groups based on their median age, and their correlation with COVID–19 were analyzed separately. It is found that BCG vaccination is not associated with COVID–19 cases and deaths for “young” and “medium” countries, but BCG vaccination remains significantly negatively correlated with both COVID–19 case and death only in “old” countries (Table 2). Importantly, in these 61 “old” countries, BCG immunization rate and median age are no longer inversely correlated (r = 0.116, p = 0.373) (Table 2). In fact, when separated by BCG coverage rates (≥50% vs <50%), among the 61 “old” countries, 36 countries with ≥50% BCG coverage have an average median age of 41.8±2.7, which is almost the same as the average median age of 41.1±3.025 for the countries with <50% BCG coverage (Figure 3C, D). Thus, after controlling for the confounding effects of age, the inverse correlation remains statistically significant between BCG vaccination and COVID–19 case (r = −0.338, p = 0.0082), and between BCG vaccination and COVID–19 death (r = −0.411, p = 0.0011), in high median-age (“old”) countries.

## Discussion

Analyses for correlation between COVID–19 deaths and eight other factors was carried out to assess which ones are important for the COVID–19 impact on different countries. The study identified median age, which is significantly associated with a country’s per capita GDP, and BCG immunization as significantly associated with both COVID–19 case and death numbers. Consistent with a correlation with per capita GDP, there is also a borderline significance in an inverse correlation between COVID–19 deaths and extreme poverty index. The borderline significance is likely due to high missing numbers for extreme poverty index (89/210 countries are missing data) that reduce the total number (n) for statistical analysis.

As an infectious respiratory disease transmitted through human contacts, it can be assumed that the higher the human density should associate with more COVID–19 cases. Social distancing works by reducing effective human density. However, it is found that COVID–19 cases and deaths are not significantly associated with population density. So most densely populated counties (or special regions), such as Monaco, Singapore, and Hong Kong, do have the highest number of COVI–19 cases or deaths, even based on per million population. Likewise, it is surprising that COVID–19 death seems not as highly correlated with factors such population density, disease control stringency, the availability of physicians and hospital beds as it does with GDP. However, there are caveats in this interpretation as certain data are incomplete, affecting statistical analysis. The stringency data are incomplete (119 countries are missing data) and do not necessarily reflect the real timely responses and control in the complex situations of countries. Furthermore, contrary to a perception that low living standard and poverty may incur high number of COVID–19 casualty, it is found that the high numbers of per million COIVD–19 cases and deaths are associated with high per capita gross domestic product (GDP), a general measure of a country’s wealth. Indeed, the top 20 countries in both total number of deaths and deaths per million include many of the world’s wealthiest nations, including the United States and most Western European countries. However, as a country’s median age is significantly associated with its per capita GDP (Table 1). It is believed that the reason the elderly are more susceptible to COVID–19 is because their immune systems have declined^13^. The correlation between GDP and COVID–19 is likely confounded by age.

BCG vaccination rates among world countries stand out as highly significantly correlated with COVID–19 morbidity and mortality, and this correlation remains significant after controlling for the confounding effect of age (Table 1, Figure 2). These results are consistent with other studies. An earlier study of analyzed data of several countries as of March 21, 2020, and has shown significant correlation between countries with universal BCG vaccination policy and reduced COVID–19 morbidity and mortality^8^. A biweekly updated report since April 2020 analyzing the daily rate of COVID–19 case and death increase has consistently shown that countries with mandated BCG vaccination policy exhibit flattened curves^7^. A report found no significant difference in COVID–19 positive test rates between two groups of young adults of about 3000 each in Israel^6^. However, one limitation is that the BCG vaccinated group (aged 39–41 years) is slightly older than the unvaccinated group (aged 35–37).

It has shown that BCG immunization can have long lasting protective effects against not only TB but also other respiratory diseases even lung cancer^9–12^. There are no proven vaccines yet against SARS-Cov2 to date, the inverse correlation between BCG vaccination and COVID–19 morbidity and mortality may suggest that BCG vaccines may offer protection against COVID–19.

## Methods

All data were from Our World in Data (OWID) based at the University of Oxford (https://ourworldindata.org/). Data as of 06–01–2020 were used for COVID–19 total deaths per million, stringency index, population density per thousand kilometer, median age, per capita gross domestic product (GDP), extreme poverty index, hospital beds per thousand people. Average rates of BCG vaccination coverage for 1-year olds for each country from 1980 to 2015, and for share of children immunized with DTP3 from 1980 to 2017 were used. For statistical analyses, Pearson’s correlation coefficient is used for measuring statistical relationships between two independent variables that are continuous and approximately normally distributed. Two-sided significance threshold was set at *p*<.05. Data processing, visualization, and statistical analyses were carried out using MATLAB®.

## Supplementary Material

Supplement

## Figures and Tables

**Table 2.** Correlation coefficient in countries grouped by median age

	Group	“Young”	“Medium”	“Old”
	N	50	75	61
	min Age	15.1	23.1	36.2
	Country	Niger	Gabon	Trinidad&Tobago
	max Age	22.9	35.7	48.2
	Country	Guatemala	Armenia	Japan
BCG vs COVID cases	r	0.095	0.065	−0.338
	p	0.514	0.58	0.0082 **
BCG vs COVID deaths	r	0.082	0.12	−0.411
	p	0.57	0.304	0.0011 **
BCG vs median Age	r	0.201	−0.128	0.116
	p	0.161	0.274	0.373

World’s 210 countries/regions were discretized into 3 groups by median age. 24 countries excluded due to lacking median age information.

** indicates p<0.01 by Pearson correlation coefficient.
