# Worldwide inverse correlation between Bacille Calmette-Guérin immunization and COVID-19 mortality

**DOI:** 10.21203/rs.3.rs-42927/v2

**Published:** 2020-09-28

**Authors:** Willis X. Li

**Affiliations:** University of California San Diego

**Keywords:** SARS-CoV-2, COVID-19, Bacille Calmette-Guérin (BCG)

## Abstract

The coronavirus disease 2019 (COVID-19) pandemic has spread to all countries in the world after more than half a year since it was first reported in late 2019, and different countries have been impacted differently. Multivariate statistical analyses were used to evaluate COVID-19 deaths and cases relative to nine other demographic and socioeconomic factors in all countries and regions of the world using data as of August 1, 2020. The factors analyzed in the study include a country’s total COVID-19 deaths and cases per million population, per capita gross domestic product (GDP), population density, virus tests per million population, median age, government response stringency index, hospital beds availability per thousand population, extreme poverty rate, Bacille Calmette-Guérin (BCG) vaccination rate, and diphtheria-tetanus-pertussis (DTP3) immunization rate. The study reveals that COVID-19 deaths per million population in a country most significantly correlates, inversely, with the country’s BCG vaccination rate, and also significantly correlates a country’s per capita GDP and median age, while COVID-19 cases per million population significantly correlate with per capita GDP and tests per thousand population. This study contributes to a growing body of evidence supporting the notion that BCG vaccination may be protective against COVID-19 mortality.

## Introduction

Since first reported in Wuhan, China, in December 2019, the COVID-19 pandemic, caused by the novel coronavirus SARS-CoV-2, has reached all countries and territories in the world more than six months later, with many countries or regions having passed the peak of their pandemics ^1^. As of August 10, 2020, there have been more than 21 million total accumulated cases and >760,000 total deaths world wide due to COVID-19, with a fatality rate of 4% ^1^. However, different countries have been impacted very differently, with about a quarter of all cases and deaths happening in the United States, while certain other countries recording much lower levels of COVID-19 cases and deaths. The differential impact of COVID-19 on different countries in the world begs the question of what factors are important in influencing COVID-19 morbidity and mortality.

As an infectious respiratory disease transmitted through human contacts, COVID-19 should have higher impact in countries with high population density, with low disease control stringency, and in countries that lack medical resources such as physicians and hospital beds, which are often associated with low per capita GDP. In addition, there have been observations of COVID-19 association with climate, BCG immunization, and various socioeconomic conditions of countries. Some of the observations were made during the early stage of the disease transmission when COVID-19 data were still accumulating, hindering the ability to draw definitive conclusions. Now more than six months have passed since COVID-19 started and all countries have been affected, it is time to examine its transmission again in order to identify factors that are most important for COVID-19 morbidity and mortality.

I have previously posted on the Research Square preprint server ^2^ that COVID-19 morbidity and mortality inversely correlate with BCG immunization by Pearson correlation coefficient analysis with data available for all countries in the world as of June 1, 2020, and that the correlation remains statistically significant after controlling for age, a factor important for COVID-19 susceptibility. The current study used the same methods to evaluate COVID-19 impact on countries worldwide as of August 1, 2020. During the two months, the world has witnessed a tripling of COVID-19 cases from 6,327,540 to 17,999,745, and a doubling of total COVID-19 deaths from 385,096 to 687,582. Despite the large increase in case and death numbers, the current analysis found that the previous results mostly hold true, suggesting that BCG vaccination may have protective effects against COVID-19.

## Results

### Disproportional distribution of COVID-19 cases and deaths in world countries

**Figure 1:**
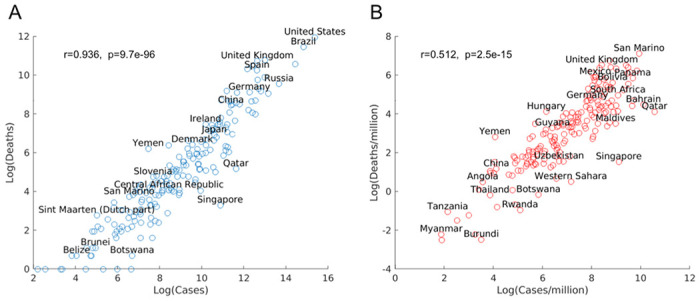
(A) Scatter plots in log scale of COVID-19 cases and deaths. (B) Scatter plots in log scale of COVID-19 cases and deaths per million. Only a subset of countries is labeled above its marker.

Although the COVID-19 pandemic has spread to all countries in the world, different countries have been impacted differently, based on data as of August 1, 2020. The U.S. recorded more than 5.5 millions cases and more than 170,000 deaths due to COVID-19, whereas some other countries these numbers are much lower, as in a scatter blot (Figure 1A). After controlling for population size, disparity for countries still remains, though a few countries with small population sizes, such as San Marino, lead the chart in COVID-19 cases and deaths per million population (Figure 1B). The case and death numbers are highly correlated, i.e., in general the higher the case number, the higher the death number in a country, although there are outliers with high case number but low death number, such as Qatar and Singapore (Figure 1). The unequal distribution of COVID-19 death and case numbers in different countries raises the question of what factors are important for the susceptibility of a population to SARS-CoV-2 infection.

### Demographic and socioeconomic factors correlated with COVID-19 impact

To understand what demographic or socioeconomic factors are important for the COVID-19 impact, two different multivariate statistical analyses were employed. First, a pairwise Pearson correlation coefficient analysis was carried out for several factors with data available for most countries. These factors include each country’s total cases and deaths per million population due to COVID-19; total numbers of SARS-CoV-2 virus tests carried out; government response stringency index, which records the strictness of ‘lockdown style’ policies that primarily restrict people’s behavior ^3^; population density (number of people per square kilometer of land); median age; per capita gross domestic product (GDP) (2019); extreme poverty index, hospital beds per thousand people; the coverage rates of Bacille Calmette-Guérin (BCG) vaccination; and diphtheria-tetanus-pertussis (DTP3) immunization rates. BCG is a vaccine against tuberculosis (TB), but there has been observations of its correlation with COVID-19 impact ^4^. DTP3 is a combined vaccine offered to young children in many, but not all countries with historical data available similarly to those of BCG immunization ^5^. Since COVID-19 death and case numbers are highly correlated, they were used separately in the following two different statistical multivariate analyses.

### Factors correlate with COVID-19 death and case in pairwise analysis

**Figure 2:**
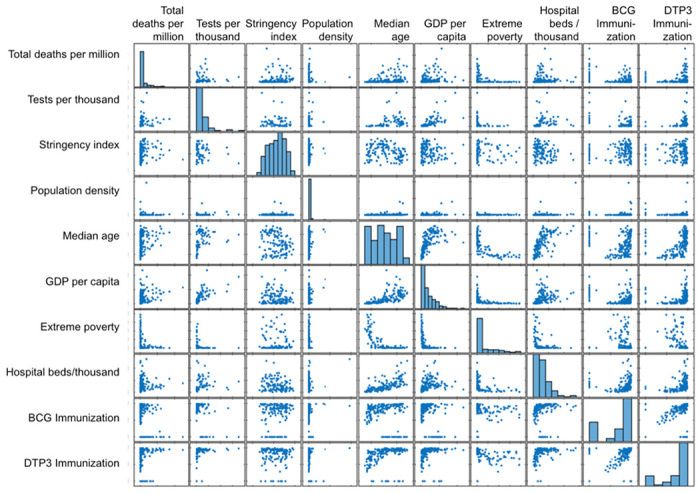
Pairwise scatter plots of the indicated independent variables. Green shaded regions indicate p<0.05 by Pearson’s correlation coefficient (see Table 1 for values).

The MATLAB^®^ function ‘corrcoef’ was first used for Pearson’s correlation coefficients for statistical relationships between independent variables. The analysis returned a matrix of correlation coefficients calculated from an input matrix whose rows are observations (210 countries and regions) and whose columns are independent variables (e.g., deaths per million, BCG rates). When these 10 factors were thus analyzed as independent variables, it is found that COVID-19 deaths per million most significantly (negatively) correlates with the country’s BCG vaccination coverage rates (r=−0.50, p=5.3e-5). COVID-19 deaths per million also significantly (positively) correlates with a country’s per capita GDP (r=0.39, p=0.0074), and with median age (r=0.30, p=0.042) (Figure 2, **Table 1**). COVID-19 death is found not significantly correlated, however, with a country’s stringency index, population density, virus tests, extreme poverty rates, hospital bed availability, and DTP3 immunization coverage (**Table 1**). Thus, fewer COVID-19 deaths were found in countries with higher rates of BCG vaccination, whereas more COVID-19 deaths are found in countries with higher per capita GDP or more elderly people.

When COVID-19 cases per million were analyzed with the same method, however, it is found that it significantly correlates only with tests per thousand and GDP per capita, suggesting that high-income countries that carried more tests have more COVID-19 cases (**Table 2**). The difference between COVID-19 case and death numbers in terms of their relationships with other demographic and socioeconomic factors begs further analysis with different methods.

### Multivariate analysis for VOVID-19 cases and deaths by linear regression

A second method used for a multivariate statistical analysis of COVID-19 case and death was a generalized linear model regression with the MATLAB^®^ function ‘glmfit’. Here COVID-19 deaths and cases per million, respectively, in each country was used as observed responses, and all other factors were used as predictors. Results from multivariate analysis with generalized linear model regression are similar to those from the above Pearson’s correlation coefficient analysis, although with interesting differences. Specifically, the most significant factor predicting COVID-19 deaths is, again, BCG immunization rates (p=3.7e-7; **Table 3**), consistent with Pearson’s correlation coefficient analysis. Also consistent with the Pearson’s correlation coefficient is that per capita GDP significantly correlates with COVID-19 deaths (p=0.016). Unlike Pearson’s correlation coefficient, the multivariate linear regression found that DTP3 immunization rates (p=0.0001), but not median age (p=0.0647), significantly correlates with COVID-19 deaths (Table 3). However, since DTP3 immunization rates positively correlates with COVID-19, the significant result is less informative, because it would suggest that DTP3 immunization promoted COVID-19 death. Thus, two different statistical methods both suggest BCG vaccination reduces COVID-19 deaths.

When COVID-19 case numbers were analyzed with multivariate linear regression, it is found that per capita GDP (p=1.75e-7) and tests per 1000 population (p=0.0093) significantly correlates with COVID-19 cases (**Table 4**), consistent with Pearson’s correlation coefficient. In addition, multivariate linear regression indicates that median age, government response stringency, and poverty levels are also correlated with COVID-19 cases (**Table 4**), unlike results from the Pearson’s correlation coefficient analysis.

BCG is a tuberculosis (TB) vaccine used in many countries with high TB prevalence ^4^. But BCG is not generally used in countries with low risks of TB infection, such as the US and most Western European countries. There have been reports of COVID-19 and BCG association with both positive and negative results ^6–8^, and currently there are ongoing clinical trials using BCG as vaccine against COVID-19 ^9,10^.

**Figure 3:**
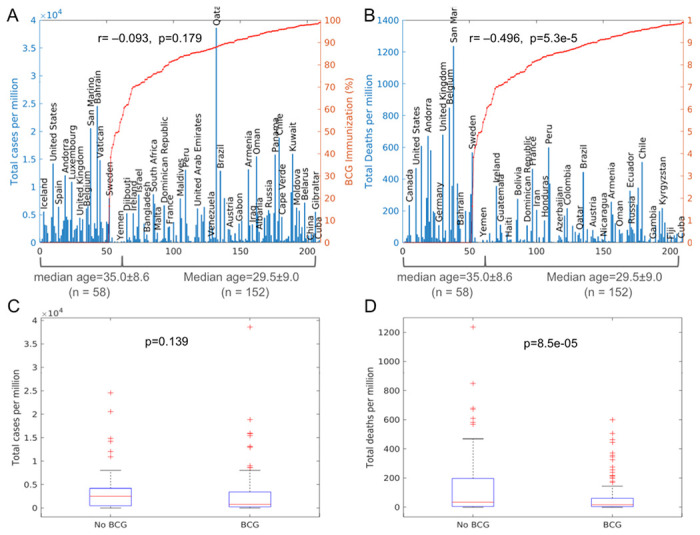
COVID-19 morbidity and mortality vs BCG immunization rates (A, B) Total COVID-19 cases and deaths per million against BCG immunization rates for all countries and regions (n=210) in the world. Only select countries are labeled. Values of r, p indicate Pearson correlation coefficient and p-value. (C, D) Boxplots of total COVID-19 cases and deaths per million for all countries and regions (n=210) in the world, grouped by BCG vaccination rate of either <50% (‘No BCG’) or ≥50% (BCG). P values indicate the levels of difference between the two groups by two-tailed Student’s t-test.

This study used BCG vaccination rates from Our World in Data ^11^, with the rates averaged for all the years with available data (1980 to 2019) for each country, and these were further verified with data from The BCG World Atlas ^12^. Although the world health organization (WHO) started recording BCG coverage data only after 1980, most countries with BCG policy started immunization before 1980 ^12^. The average BCG vaccination rates over all the years show highly significant inverse correlation with COVID-19 deaths by two statistics methods (**Table 1, 3**). Indeed, when countries are sorted by mean BCG immunization rate, it can be seen that the high COVID-19 deaths occurred more in countries with low BCG vaccination rates (Figure 3).

The world countries’ BCG coverage rates show a dichotomous pattern, and there is a steep drop from high coverage rates (nearly 100%) to no coverage (0%) (Figure 3A B). Very few counties have a BCG coverage rate around 50% (a total of 9 countries have BCG rates from 40% to 60%). When countries were separated by BCG coverage rates into two groups of BCG rates≥50% (denoted as “BCG”) and BCG rates <50% (denoted as “No BCG”), the two groups of countries show significantly different mean deaths, but not significantly different mean cases (Figure 3C,D). This is in agreement with results from both Pearson’s correlation coefficient analysis and multivariate linear regression.

In summary, factors affecting COVID-19 cases and deaths were analyzed using Pearson’s correlation coefficient and multivariate linear regression. Both statistics methods indicate that COVID-19 deaths most significantly correlate with a country’s BCG immunization rate, while the number of coronavirus positive cases correlate with the country’s resourcefulness and testing capabilities.

### Correlation between COVID-19 death and GDP may be due to age

A single factor identified by two statistical methods that significantly correlates with both COVID-19 cases and deaths is per capita GDP (**Table 1 - 4**). It has been reported that COVID-19 affects older people more than younger people ^13^. Indeed, worldwide COVID-19 deaths significantly correlate with the median age of nations (r=0.30, p=0.042) by Pearson’s correlation (Figure 2, **Table 1**). On the other hand, the median age of a country highly significantly correlates with its per capita GDP (r=0.64, p=1.93e-6). Thus a plausible explanation is that high per capita GDP leads to longer lifespan, thereby raising the median age of a country. In other words, the higher case and death numbers in wealthy countries were likely due to the higher age as a confounding factor in those countries.

### BCG vaccination and COVID-19 death inversely correlate in countries with high median age

Since age may be a significant confounder in the correlation between GDP and COVID-19, is age similarly a confounding factor in the correlation between BCG vaccination and COVID-19? Correlation coefficient analysis shows that BGC vaccination does not significantly correlate with a country’s median age (r= −0.24, p=0.107; **Table 1**), although there appears to be a trend that countries with lower BCG vaccination rates may have a higher median age. This raised the question of whether the higher COVID-19 case and death numbers in countries lacking BCG vaccination can be confounded by their having more elderly population.

To reduce the possible confounding effects of age, propensity score matching for age was used to further evaluate the correlation between BCG vaccination and COVID-19. To this end, all countries were divided into three groups by median age. The median ages of the world’s 210 countries and regions range from 15.1 (Niger) to 48.2 (Japan) (**Table 5**). These countries were discretized into “young”, “medium”, and ‘old” groups based on their median age, and their correlation with COVID-19 were analyzed separately in age-matched subgroups. It is found that BCG vaccination is not associated with COVID-19 cases and deaths for “young” and “medium” aged countries, but BCG vaccination remains significantly negatively associated with both COVID-19 case and death only in “old” countries (**Table 5**). Importantly, in these 61 “old” countries, BCG immunization rate and median age are no longer inversely correlated (r=0.116, p=0.373) (**Table 5**).

**Figure 4:**
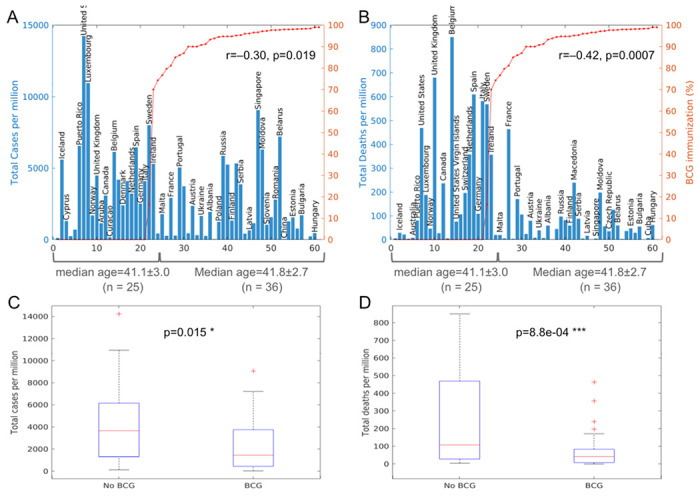
COVID-19 morbidity and mortality vs BCG immunization in ‘old’ countries (A, B) Total COVID-19 cases and deaths per million against BCG immunization rates for high median age (“old”) countries and regions (n=61). r and p are from Pearson’s correlation coefficient analysis. (C, D) Boxplots of total COVID-19 cases and deaths per million ‘old” all countries in the world (n=61), grouped by BCG vaccination rate of either <50% (‘No BCG”) or ≥50% (BCG). P values indicate the levels of difference between the two groups by two-tailed Student’s t-test.

When separated by BCG coverage rates (≥50% vs <50%), among the 61 “old” countries, 36 countries with ≥50% BCG coverage have an average median age of 41.8±2.7, which is almost the same as the average median age of 41.1±3.025 for the countries with <50% BCG coverage (Figure 4). Thus, after controlling for the confounding effects of old age by propensity score matching, the inverse correlation remains statistically significant between BCG vaccination and COVID-19 case (r=−0.30, p=0.019), and between BCG vaccination and COVID-19 death (r=−0.42, p=0.0007), in high median-age (“old”) countries. Thus, BCG vaccination may reduce COVID-19 mortality in high median age countries.

## Discussion

Analyses for correlation between COVID-19 deaths and nine socioeconomic factors was carried out in order to assess which factors are important for the COVID-19 impact on world countries. The study identified BCG immunization as most significantly correlated, inversely, with COVID-19 mortality eight months into the pandemic, and this correlation remains significant after controlling for the confounding effect of age. Other factors significantly correlate with COVID-19 deaths a country’s per capita GDP and median age. These two factors highly significantly correlate with each other, and age, which is known to affect COVID-19 susceptibility, is likely a confounder for the effects of per capita GDP.

As an infectious respiratory disease transmitted through human contacts, it can be assumed that the higher the human density should result in more COVID-19 cases and deaths. Social distancing works by reducing effective human density. However, this study found that COVID-19 cases and deaths do not significantly correlate with population density. So most densely populated counties or regions, such as Monaco, Singapore, and Hong Kong, do not have the highest number of COVID-19 cases or deaths, even based on per million population. Likewise, it was surprising that COVID-19 deaths did not significantly correlate with factors such as government disease control stringency and the availability of hospital beds. However, there are caveats in this interpretation as certain data are incomplete, affecting statistical analysis. In particular, the policy stringency index data are incomplete (119 countries are missing data) and do not necessarily reflect the real timely responses and control in the complex situations of different countries.

Contrary to the perception that low living standard and poverty may incur high number of COVID-19 casualty, it is found that the high numbers of per million COIVD-19 cases and deaths correlate with high per capita GDR a general measure of a country’s wealth. Indeed, the top 20 countries in both total number of deaths and deaths per million include many of the world’s wealthiest nations, including the United States and most Western European countries. However, as a country’s median age is significantly correlated with its per capita GDP. It is believed that the reason the elderly are more susceptible to COVID-19 is because their immune systems have declined ^14^. Thus, the correlation between GDP and COVID-19 is likely confounded by age.

Although it is also argued that low GDP countries may lack testing capabilities, thus underestimating COVID-19 impacts. While this may be true in the early days or months of the pandemic, testing ability has increased to levels comparable to high-income countries ^15^. This study found COVID-19 deaths not significantly correlated with total tests carried out by countries, although understandably COVID-19 cases significantly correlates with testing.

To assess if other types of vaccination also influence COVID-19 deaths, immunization against diphtheria tetanus toxoid and pertussis (DTP3) was analyzed. DTP3 immunization is another long running vaccine program adopted non-uniformly by different countries, and broad immune benefits have been reported for both BCG and DTP3 ^16,17^. However, unlike BCG, no significant correlation was found between DTP3 immunization and COVID-19 death by Pearson’s correlation (r=0.24, p=0.121; Table 1). Although DTP3 did show significant correlation with COVID-19 when analyzed by multivariate linear regression, this correlation would indicate DTP3 vaccination increases CVOID-19 deaths, because the coefficient is positive, as mentioned earlier. Thus, BCG vaccination might have specific effects on reducing COVID-19 deaths.

There have been other studies recently on BCG and COVID-19 correlation, with different conclusions. Miller et al analyzed data of several countries as of March 21, 2020, and found a significant correlation between countries with universal BCG vaccination policy and reduced COVID-19 morbidity and mortality ^8^. Berg et al have published a biweekly updated report since April 2020 analyzing the daily rates of COVID-19 case and death increase, and have shown that countries with mandated BCG vaccination policy consistently exhibit flattened curves (slower increases) ^7^. A recent study by Escobar et al carried out detailed population comparisons, e.g., between former East and West Germany, and have found significant correlation BCG immunization and reduced COVID-19 deaths especially in European countries ^18^. These studies are consistent with the current study. There are also reports, however, showing the opposite. For example, Hamiel et al found no significant difference in COVID-19 positive test rates between two groups of young adults of about 3000 each in Israel ^6^. It is noted that the BCG vaccinated group (aged 39-41 years) is slightly older than the unvaccinated group (aged 35-37), and the study examined only SARS-CoV2 infection not mortality. Asahara analyzed COVID-19 cases among passengers boarding the *Diamond Princess* cruise ship and found no significant correlation between citizens from countries with mandatory BCG immunization and those with no such policy ^19^. Thus, further investigation is clearly needed to establish a causal relationship, if any, between BCG vaccine and COVID-19 mortality. Nonetheless, a growing consensus seems to suggest that BCG vaccination may help reduce COVID-19 mortality.

BCG vaccine was initially developed in 1921 in France as attenuated live strains of *Mycobacterium bovis*, but the strains used in different countries have since shown difference in eliciting human immune reactions ^20^. This study did not attempt to distinguish among different strains of BCG vaccines. Future studies may compare different BCG strains toward mechanistic understanding if and how BCG vaccine might be used protect against diseases other than TB. Indeed, it has shown that BCG immunization can have long lasting protective effects against not only TB but also other respiratory diseases including even lung cancer ^9,10,16,17^ . There are no proven vaccines yet against SARS-CoV-2 to date. If the proposed BCG protection hypothesis holds true, BCG could be used a substitute if COVID-19 vaccines cannot be established. Ongoing trials of COVID-19 vaccines may benefit by comparing with BCG for effects against SARS-CoV-2.

As with all epidemiological studies, the current study has limitations. Notably, COVID-19 data at the country levels may not fully reflect local provincial situation and may thus represent a sampling bias in statistical studies. Future studies should be at individual levels to alleviate sampling bias. Nonetheless, the inverse correlation between BCG vaccination and COVID-19 mortality found in this and other mostly epidemiological studies suggest that BCG vaccines may offer protection against COVID-19.

## Methods

All data were from Our World in Data (OWID) based at the University of Oxford (https://ourworldindata.org/) ^1^. Data as of 08-01-2020 were used for COVID-19 total deaths per million, stringency index, population density per thousand kilometer, median age, per capita gross domestic product (GDP), extreme poverty index, hospital beds per thousand people. Average rates of BCG vaccination coverage for 1-year olds for each country from 1980 to 2019, and for share of children immunized with DTP3 from 1980 to 2018 were also from Our World in Data (OWID) ^11^. BCG coverage rates were verified using data from The World BCG Atlas (http://www.bcgatlas.org/index.php) ^12^.

For statistical analyses, Pearson’s correlation coefficient was used for measuring statistical relationships between two independent variables that are continuous and approximately normally distributed. The MATLAB^®^ function *‘corrcoef’* was used for multivariate analysis, which returns a matrix of correlation coefficients calculated from an input matrix whose rows are observations (countries) and whose columns are variables (e.g., deaths per million, BCG rates). Two-sided significance threshold was set at *p*<.05. Data processing, visualization, and statistical analyses were carried out using MATLAB^®^ Online (R2020a; The MathWorks Inc. 2020).
