# Supplementary material for: Worldwide inverse correlation between Bacille Calmette-Guérin immunization and COVID-19 morbidity and mortality
Source: Res Sq. 2020 Jul 16:rs.3.rs-42927. Preprint. [Version 1] doi: 10.21203/rs.3.rs-42927/v1 (PMC7373138; doi:10.21203/rs.3.rs-42927/v1)
Supplement: Supplementary file 1 [file table1.docx]

Table 1. Pearson's correlation coefficients and *p*-values for different pairs of variables

	Total deaths per million	Stringency index	Population density	Median age	GDP per capita	Extreme poverty	Hospital beds / thousand	BCG Immuni-zation	DTP3 Immuni-zation	*r/p*
Total deaths per million	1	0.0075488	0.070911	0.4803	0.55064	-0.27713	0.08517	-0.63424	0.23755	*r*
	1	0.95894	0.62826	0.00047819	4.1422e-05	0.053882	0.56066	9.9298e-07	0.10027	*p*
Stringency index	0.0075488	1	0.12764	-0.056958	-0.17008	-0.13327	-0.16099	0.17754	-0.069177	*r*
	0.95894	1	0.38211	0.69748	0.24265	0.36131	0.26914	0.22231	0.63671	*p*
Population density	0.070911	0.12764	1	0.20177	0.096543	-0.12687	0.28098	-0.038317	0.067435	*r*
	0.62826	0.38211	1	0.16444	0.50932	0.38503	0.050505	0.79379	0.64524	*p*
Median age	0.4803	-0.056958	0.20177	1	0.66176	-0.79161	0.58521	-0.26124	0.67146	*r*
	0.00047819	0.69748	0.16444	1	2.2503e-07	1.2728e-11	1.0039e-05	0.06982	1.2849e-07	*p*
GDP per capita	0.55064	-0.17008	0.096543	0.66176	1	-0.50001	0.41498	-0.64932	0.46838	*r*
	4.1422e-05	0.24265	0.50932	2.2503e-07	1	0.00025408	0.0030288	4.486e-07	0.00068862	*p*
Extreme poverty	-0.27713	-0.13327	-0.12687	-0.79161	-0.50001	1	-0.44224	0.12808	-0.60627	*r*
	0.053882	0.36131	0.38503	1.2728e-11	0.00025408	1	0.0014648	0.38045	3.8968e-06	*p*
Hospital beds/thousand	0.08517	-0.16099	0.28098	0.58521	0.41498	-0.44224	1	0.015345	0.45755	*r*
	0.56066	0.26914	0.050505	1.0039e-05	0.0030288	0.0014648	1	0.91666	0.00094828	*p*
BCG Immunization	-0.63424	0.17754	-0.038317	-0.26124	-0.64932	0.12808	0.015345	1	0.0065053	*r*
	9.9298e-07	0.22231	0.79379	0.06982	4.486e-07	0.38045	0.91666	1	0.96462	*p*
DTP3 Immunization	0.23755	-0.069177	0.067435	0.67146	0.46838	-0.60627	0.45755	0.0065053	1	*r*
	0.10027	0.63671	0.64524	1.2849e-07	0.00068862	3.8968e-06	0.00094828	0.96462	1	*p*

Pearson's correlation coefficient and p-value for the indicated independent variables compared in pairwise. Two-sided significance threshold was set at $p<.05$ (shaded in green).
